# When and How to Use MIC in Clinical Practice?

**DOI:** 10.3390/antibiotics11121748

**Published:** 2022-12-03

**Authors:** Sophie Magréault, Françoise Jauréguy, Etienne Carbonnelle, Jean-Ralph Zahar

**Affiliations:** 1 Department of Pharmacology, AP-HP, Groupe Hospitalier Paris Seine Saint-Denis, F-93140 Bondy, France; 2 Université Sorbonne Paris Nord and Université Paris Cité, Inserm, IAME, F-93000 Bobigny, France; 3 Department of Clinical Microbiology, AP-HP, Groupe Hospitalier Paris Seine Saint-Denis, 93009 Bobigny, France

**Keywords:** microbiology, minimal inhibitory concentration, pharmacology, PK/PD parameters, TDM-guided dosing adjustment, clinical practice

## Abstract

Bacterial resistance to antibiotics continues to be a global public health problem. The choice of the most effective antibiotic and the use of an adapted dose in the initial phase of the infection are essential to limit the emergence of resistance. This will depend on (i) the isolated bacteria and its resistance profile, (ii) the pharmacodynamic (PD) profile of the antibiotic used and its level of toxicity, (iii) the site of infection, and (iv) the pharmacokinetic (PK) profile of the patient. In order to take account of both parameters to optimize the administered treatment, a minimal inhibitory concentration (MIC) determination associated with therapeutic drug monitoring (TDM) and their combined interpretation are required. The objective of this narrative review is thus to suggest microbiological, pharmacological, and/or clinical situations for which this approach could be useful. Regarding the microbiological aspect, such as the detection of antibiotic resistance and its level, the preservation of broad-spectrum β-lactams is particularly discussed. PK-PD profiles are relevant for difficult-to-reach infections and specific populations such as intensive care patients, cystic fibrosis patients, obese, or elderly patients. Finally, MIC and TDM are tools available to clinicians, who should not hesitate to use them to manage their patients.

## 1. Introduction

The increasing resistance of bacteria to antibiotics is a major worldwide public health problem, particularly considering the association of increased mortality and length of hospital admission for patients with multidrug-resistant (MDR) bacterial infections [1]. It is urgent to assess ways to reduce the global spread of bacterial resistance and to select the most effective antibiotic during the initial phase of infection, particularly when resistance affects the most broad-spectrum agents such as carbapenems or, recently, last-line therapies such as β-lactams/β-lactamase inhibitor (BLBLI) combinations and cefiderocol [2,3].

Before targeting an optimal antibiotic therapy, an empirical treatment is administered, and the previous collection of a microbiological sample helps choose the most effective treatment. Among the microbiological results, antibiotic susceptibility testing (AST) is currently based on testing the ability of an antibiotic to inhibit bacterial growth in vitro under standardized experimental conditions. Antibiotic susceptibility is mostly measured by the diameter (in mm) of the zone of bacterial growth inhibition around an antibiotic-containing disk versus the tested pathogen. AST reflects the responsible pathogen’s susceptibility or resistance to diverse antibiotics, leading to the selection of a clinically effective antimicrobial treatment. For most bacterial infections, AST measured using a diameter is sufficient. However, in the case of severely ill patients with chronic infections previously treated with many broad-spectrum antibiotics, more accurate information is needed to facilitate the selection of an optimal antibiotic regimen. 

The minimal inhibitory concentration (MIC) may help to choose the most appropriate treatment. It defines in vitro the levels of susceptibility or the resistance of specific bacterial strains to a targeted antibiotic [4]. However, it is difficult to predict the clinical outcome of an infection only based on the MIC value. Several pharmacokinetic and pharmacodynamic (PK/PD) parameters have been associated more precisely with patient or infection outcomes for some antibiotics and PK/PD measurements help to optimize antibiotic therapy and to minimize the emergence of resistance [5,6]. Moreover, MIC may be determined in other clinical situations in severely ill patients (especially in cases of renal injury or augmented renal clearance [5,7]) or in the case of difficult-to-reach sites of infection (i.e., endocarditis or meningitis [8,9]). The aim of this article is to discuss the microbiological and pharmacological points of view regarding a clinical situation in order to propose specific cases where MIC determination and therapeutic drug monitoring (TDM) may be beneficial for the patient.

## 2. MIC and Its Microbiological Indications

Classic AST, e.g., critical diameter measurement, raises problems in some conditions, leading to the determination of the MIC value. We will detail below the methods that can be used, their relevance, and their microbiological indications. 

### 2.1. MIC Determination Methods

Two main methods are used to determine MIC [10]. Broth microdilution (BMD) is a method in which containers are filled with identical volumes of inoculated broth and identical volumes of an antibiotic solution, but incrementally (usually geometrically) increasing concentrations of the antibiotic and a defined inoculum. The results are recorded as the lowest concentration of antimicrobial agent that inhibits the visible growth of a microorganism, MIC, expressed in mg/L or µg/mL. Agar dilution involves the incorporation of an antibiotic in solid or semi-solid agar media in a geometrical progression of concentrations and the application of a defined bacterial inoculum to the surface. Its purpose is the determination of the lowest concentration that inhibits bacterial growth, namely MIC [10]. The responsible bacteria are susceptible or resistant if the antibiotic MIC is below or above the clinical breakpoint cut-off, respectively. The “European Committee on Antimicrobial Susceptibility Testing” (EUCAST) annually updates the clinical breakpoint tables for the interpretation of MICs and zone diameters [11]. 

### 2.2. Relevance and Microbiological Indication of MIC Determination

Classic AST raises problems in certain conditions, leading to the need to perform a specific MIC measurement. We will detail below the microbiological indications, their limits, and the methods that can be used. In addition, the microbiological relevance of MIC determination is summarized in Table 1.

#### 2.2.1. Agar Diffusion Method Is Inappropriate for Some Antibiotics

For some antibiotics, the agar diffusion method (disc and gradient strip) does not allow for the interpretation of the susceptibility of the tested microorganisms. This is due to their poor diffusion in a solid medium (colistin) or the need for particular chemical conditions (lipopeptides such as daptomycin or dalbavancin) [11]. Therefore, the EUCAST guidelines recommend the use of a BMD method to determine MIC [12]. 

#### 2.2.2. Absence of Detection of the Resistance Level to β-Lactams

For some microbiological species, it is difficult in the case of resistance to the usually tested antibiotic to define the resistance mechanism and to specify the optimal treatment choices. This is the case for β-lactam resistance in *Streptococcus pneumoniae* resistant to oxacillin (e.g., suspected of reduced susceptibility to penicillin) and *Haemophilus influenzae* resistant to aminopenicillins. 

In the first case, for *S. pneumoniae*, resistance is associated with alterations in penicillin-binding proteins (PBPs) that reduce the binding affinity of the antibiotic to PBPs [13]. As the *S. pneumoniae* genome encodes six PBPs and each β-lactam inhibits different PBPs, the modification of PBPs leads to an increase in the MICs of all β-lactams, but the extent of this increase varies according to the antibiotic [14]. 

Whereas in the second case (*H. influenzae*), two main mechanisms of amino penicillin (AMP) resistance lead to reduced susceptibility to this antibiotic class: either by the production of a β-lactamase, or by alteration of PBP3 [15]. In addition, the β-lactam MIC differs according to the degree of alteration of PBP3. It may be difficult using the disc method to distinguish β-lactamase-negative ampicillin susceptible (BLNAS) strains from β-lactamase-negative ampicillin resistance (BLNAR) strains, because most discs contain high concentrations of β-lactams [16]. 

Determination of the MIC of β-lactams to define the most appropriate treatment will be more justified when a practitioner is dealing with severe or invasive infections (such as bacteremia or meningitis), clinical failure, and/or an isolate suspected of reduced susceptibility to penicillin (*S. pneumoniae)* or AMP (*H. influenzae)*. EUCAST guidelines recommend testing of the β-lactams of interest, particularly in these cases [12].

#### 2.2.3. Detection of Low-Level Antibiotic Resistance 

Fluoroquinolone resistance in *Salmonella* is mainly caused by chromosomal mutations in the quinolone resistance-determining regions (QRDRs) of the topoisomerase genes [17] that lead to resistance to nalidixic acid (MIC > 16 mg/L) and higher MIC values for ciprofloxacin (at least 0.12 mg/L). Moreover, resistance may be associated with other diverse mechanisms of resistance, such as plasmid-mediated quinolone resistance (PMQR) mechanisms that result in reduced susceptibility to ciprofloxacin (MIC of 0.125 to 1.0 mg/L), but only a modest or no increase in susceptibility to nalidixic acid [17]. Indeed, PMQR mechanisms are clinically relevant because patients infected with *Salmonella typhi* or non-typhoidal *Salmonella* isolates with ciprofloxacin MICs of 0.125 to 1.0 mg/L have more treatment failures and longer times to fever clearance than patients with isolates fully susceptible to ciprofloxacin (MICs < 0.06 mg/L) [18]. Thus, using the disk method, the ciprofloxacin disk fails to detect this low-level resistance [19]. 

#### 2.2.4. MIC Creep

The first option for the treatment of invasive methicillin-resistant *Staphylococcus aureus* (MRSA) infections is vancomycin, which continues to be the reference standard approach in this context. However, an increasing number of MRSA isolates with high MICs, within the susceptible range (vancomycin MIC creep), are being reported worldwide. It has been reported that the efficacy of vancomycin therapy is contingent upon a target AUC_0–24_/MIC ratio of ≥400 (see next section on PK/PD indices) [20]. Nevertheless, AUC values greater than 600 mg.h/L are also associated with a higher risk of acute kidney injury, making it nearly impossible to safely and effectively treat microorganisms with vancomycin MICs > 1 mg/L [21]. Moreover, a few studies have reported poorer clinical outcomes and increased mortality associated with vancomycin MIC creep [22,23]. Divergent studies of this phenomenon have been reported in the literature [20,21] and the determination of vancomycin MIC in challenging situations will be discussed in terms of improving PK-PD target selection. Currently, EUCAST guidelines recommend the use of a reference laboratory to confirm the GISA or hetero GISA character of an *S. aureus* isolate if the vancomycin and/or teicoplanin MIC is >1 mg/L, using the BMD method [24]. 

#### 2.2.5. Preservation of Broad-Spectrum Antibiotics

Piperacillin-Tazobactam/Cephalosporins and ESBL-Producing Strains

Carbapenems have been considered as the treatment of choice for severe infections caused by extended spectrum β-lactamase (ESBL)-producers [25,26]. The increasing worldwide incidence of ESBL-related infections has led to the increased use of carbapenems, leading to selection pressure for carbapenem resistance [27,28]. Therefore, to avoid the use of carbapenems, several authors have suggested the use of antibiotics that are active with regard to AST, despite the fact that they are hydrolyzed by the ESBL enzyme. Thus, EUCAST guidelines recommend reporting ESBL-producing strains as resistant to all penicillins, but as susceptible to BLBLI combinations or third-generation cephalosporins (3GC) when they are active on AST [29]. In addition, when susceptible, the use of β-lactam/β-lactamase inhibitor (BLBLI) combinations or cephalosporins has been proposed as an alternative to carbapenems [30]. Despite controversies [31,32], success when using these antibiotics depends on several factors, including the microbial species, the site of infection [33] and the MIC [34]. Studies show that success was more frequent in cases of urinary or biliary tract infections related to *Escherichia coli* [33] and that mortality was lower for isolates with an MIC ≤ 4 mg/L for BLBLI than for isolates with a higher MIC [31,34]. However, the use of 3CG s is more rarely possible as many ESBL-producing isolates are resistant, and such antibiotics should be limited to *Escherichia coli* strains or *Klebsiella pneumoniae*-related infections with MICs below 1 mg/L [35]. In conclusion, although alternatives have been studied, carbapenems remain the drugs of choice against ESBL-positive strains [36].

#### 2.2.6. Therapy for Carbapenemase-Producing Enterobacterales (CPE)-Related Infections

The release of new antibiotics has opened up many new possibilities in the treatment of CPE-related infections [36]. Indeed, until the arrival of the new BLBLI, the cornerstone of treatment for CPE-related infections was a combination of antibiotics [37]. In addition, it has been demonstrated that these new associations (e.g., aztreonam with ceftazidime/avibactam) are effective, regardless of the mechanisms of resistance [36,38,39]. Although each molecule’s MIC is independently high, the β-lactamase inhibitor will be responsible for restoring susceptibility to β-lactams. In case of associations, a simple way to determine MICs is based on the Etest strip superposition method which has been shown to be particularly effective for aztreonam/inhibitor combinations [40]. However, the lack of availability of these new antibiotics in low- and middle-income countries highlights the possibility of using carbapenems in combination or as a therapeutic option in patients with infections using CPE isolates with meropenem MIC ≤ 8 mg/L or the combination of ertapenem with meropenem in the case of infections related to *Klebsiella pneumoniae* carbapenemase (KPC)-producing bacteria [36,40]. 

## 3. Relevance of PK/PD Indices and TDM 

### 3.1. PK-PD Indices

The aim of TDM is optimization of the dose regimen to maximize treatment efficacy while reducing the risk of toxicity and the emergence of bacterial resistance [5]. However, paradoxically, while the individual PK characteristics of the patient are generally taken into account, the PD data of the offending organism are often little or not considered, and the targets used are most often based on the EUCAST clinical breakpoint or using MIC distributions such as epidemiological cut-off values (ECOFF) [5,6,41].

These targets are based on the classically described PK-PD indices with (i) time-dependent antibiotics for which the index of interest is the percentage of time the free drug concentration remains above the MIC (%*f*T_>MIC_), (ii) concentration-dependent antibiotic in which it is the ratio of maximum antibiotic concentration (C_max_) to MIC (C_max_/MIC), and (iii) mixed-effect antibiotics in which %*f*T_>MIC_ and C_max_/MIC are of interest and are characterized by the ratio of the free drug area under the concentration–time curve during a 24-h period to MIC (*f*AUC_0-24_/MIC) [42,43]. The PK-PD indices of the most commonly used antibiotics are presented in Table 2.

An a priori dose regimen adaptation—before TDM—should follow recommendations for a specific population and clinical setting, and one way to determine the initial dosage is to review the probability of target attainment (PTA) corresponding to our specific situation [58]. PTA is based on Monte Carlo simulations and corresponds to the probability that at least a specific value of a PD index is achieved for a given MIC [43]. More and more PK-PD studies are comparing a dose regimen and/or administration modality with therapeutic success, for example for β-lactam antibiotics [59,60,61,62,63], fluoroquinolones [64], and aminoglycosides [65]. However, as these probabilities are deduced from a population PK model, this implies that the patient concerned corresponds to this specific population, and that extrapolation of PK parameters from one population to another (e.g., data from neutropenic oncology patients extrapolated to intensive care patients) is not possible.

### 3.2. Relevance of MIC Determination to Perform TDM 

A posteriori adaptation using concentration measurement is thus recommended for many antibiotics [5]. However, how does knowledge of MIC improve PK-PD target selection rather than using the usual breakpoints? Especially in cases where there is an acute risk of toxicity (e.g., with antibiotics with a narrow therapeutic window) or, conversely, a risk of underexposure due to PK (such as increased renal clearance) or PD (e.g., hard-to-reach infectious site) characteristics?

#### 3.2.1. Situations at Risk of Toxicity

Toxicity due to overdosage may occur for antibiotics with a narrow therapeutic range and/or in the case of organ failure, especially the kidney, as most antibiotics are renally cleared [7]. In the latter case, a simple adjustment of the dose regimen and repeated TDM can limit the risks of toxicity. However, in the case of a narrow therapeutic window, flexibility is reduced, and a low MIC may help. Available not-to-exceed concentration thresholds for the most used antibiotics are indicated in Table 2. 

For β-lactam antibiotics, cefepim is of the greatest concern for neurological toxicity, with a threshold of C_min_ < 22 mg/L if used by intermittent infusion and a steady-state concentration (C_ss_) < 35 mg/L if used by continuous infusion [44,45]. So, for example, in the case of *Pseudomonas aeruginosa* infection, the EUCAST clinical breakpoint is equal to 8 mg/L, implying a therapeutic target between 32 mg/L (because of a PK-PD target of 100% *f*T_>4x MIC_) and 35 mg/L if used in continuous infusion [6,11]. In this case, the information provided by the MIC of the offending organism, if below the EUCAST clinical breakpoint, would allow us to target lower concentrations, thus limiting the risk of toxicity.

Similar concerns exist for colistin and *Enterobacterales*, for which the concentrations should be greater than 2 mg/L, but less than 2.4 mg/L when considering the clinical breakpoint [29,57,66]. 

#### 3.2.2. Situations at Risk of Under-Exposure

##### Populations at Risk of Drug Under-Exposure 

The use of recommended doses may be associated with sub-therapeutic exposure, particularly in intensive care patients [67]. A specific PK profile has been described in this population with a very high inter-individual variability, an increased volume of distribution, and potentially augmented renal clearance (ARC) [7,67]. ARC is defined by a creatinine clearance (CL_CR_) higher than 130 mL/min/1.73 m², occurs in 20% to 65% of ICU patients, and corresponds to an increase in renal blood flow that often arises in septic shock [7,58,68]. It causes a significant increase in the clearance of renally eliminated antibiotics leading to a risk of not reaching PK-PD targets. For example, the population PK of piperacillin was investigated in a study in 110 ICU patients [69]. This study indicated that for patients with CL_CR_ > 90 mL/min, a high dose of 24 g per day by continuous infusion did not achieve 100% *f*T_>4x MIC_ against bacteria with MIC ≥ 4 mg/L [69]. In this population, use of a higher daily dose and/or extended intermittent infusions or continuous infusion is the best way to increase the probability of PK-PD target attainment [7]. However, again, a low MIC would avoid significant dose increases by using up to twice or more of the recommended dose regimen.

##### Difficult-to-Reach Sites of Infections

TDM is mainly performed on plasma samples and may not reflect antibiotic exposure at the site of infection, particularly in cases of endocarditis, mediastinitis, central nervous system infections, pneumopathy, or infections on prosthetic material [6]. It is thus important to consider the diffusion of antibiotics in the tissues concerned and to adapt the plasma target accordingly [70,71]. For example, when considering lung diffusion, the criterion of interest is the ratio of epithelial lining fluid (ELF) concentration to plasma concentration (ELF/plasma ratio). Antibiotics are then classified according to their diffusion with (i) ELF/plasma ratio < 1: vancomycin, meropenem, and piperacillin; (ii) ELF/plasma ratio ~1: cefepime and linezolid; and (iii) ELF/plasma ratio > 1: fluoroquinolones and azithromycin [70,71]. Antibiotics with the highest diffusion will be thus be useful for organisms with a high MIC.

Direct measurement at the site of infection, when possible, is also interesting and allows for a direct comparison of an antibiotic concentration with the MIC of the offending organism. This has been described, for example, for cefotaxime in peritoneal fluid, meropenem in cerebrospinal fluid, and colistin in drainage fluid in the context of prosthesis infection [61,72,73].

## 4. Discussion

For a long time, therapeutic monitoring of antibiotic prescriptions was limited to treatments with a narrow therapeutic index (aminoglycosides and glycopeptides) in order to avoid any treatment-related toxicity. Over the last 20 years, numerous publications have suggested the importance and value of monitoring plasma antibiotic levels to cure the infected patient and avoid the emergence of resistance that may be responsible for therapeutic failure or recurrence. Numerous populations at risk of antibiotic underdosing have been described in the literature, both in the ICU [74,75,76] and outside the ICU [65]. In addition, the emergence of resistance within microbial species, including previously susceptible strains, has increased interest in antibiotic dosing and in adapting dosing and administration methods. Thus, several studies [20,48,50,67,77,78] have emphasized the importance of administration methods in the outcome of infected patients. 

However, few publications have evaluated the importance of MIC determination in the outcome of infected patients, even though the antibiotic dosage cannot theoretically be interpreted for a given patient in the absence of MIC. Indeed, difficulty in obtaining the MIC, uncertainties related to the interpretation of this test, and delays in obtaining the results are, in our opinion, the three elements that make the use of this test difficult in clinical practice. 

Thus, most of the studies carried out to date and demonstrating or suggesting the interest of the MIC determination have been based essentially on a “theoretical” MIC, generally the highest MIC for the chosen antibiotic and found in the species responsible for the infection. This choice, which currently seems to be accepted by various authors, does, however, run the risk of individual toxicity and of collective antibiotic overconsumption. Therefore, the question of the interest of MIC determination in clinical practice is the same as the question of the right prescription, i.e., the right antibiotic dose that best responds to the PK/PD data, for a given microbial species, in a particular tissue infection and in a patient with his/her own specificities. It is thus obvious that many infections will not require antibiotic dosing or MIC determination, provided that the antibiotic administered at the usual doses diffuses to the infection site at sufficient concentrations and below toxic concentrations, concentrations that are equivalent to and exceed the PK/PD parameters (see above) required to cure and avoid the emergence of antibiotic resistance. 

**Figure 1:**
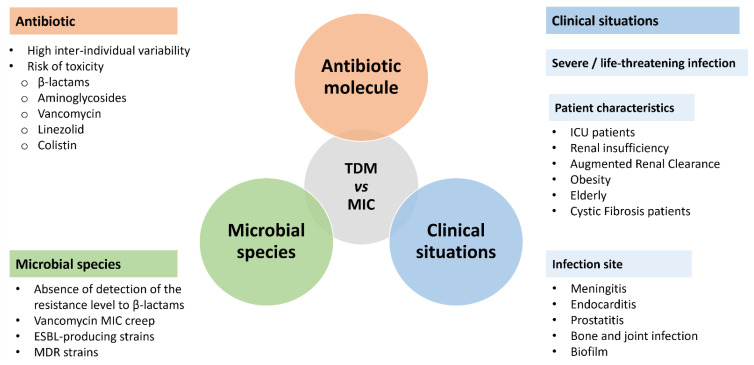
Microbiological, pharmacological, and clinical situations where an interpretation of the antibiotic concentration in relation to the MIC determination would be useful. TDM: therapeutic drug monitoring; MIC: minimal inhibitory concentration; ESBL: extended spectrum β-lactamase; MDR: multidrug-resistant.

Nevertheless, there are still many clinical situations that do not meet the above-mentioned characteristics and for which MIC determination should be considered. Indeed, the literature data lead us to propose a strategy that mainly includes the three main actors: the infectious site, the microbial species, and the antibiotic (Figure 1). Moreover, certain clinical situations seem to be more at risk of therapeutic failure due to the low diffusion of some antibiotics, but the risk cannot be conceived for all microbial species or for all antibiotics. Thus, for example, a brain or meningeal infection, where most antibiotics are poorly distributed, due to MRSA [79] or *S. pneumoniae* [80], with reduced susceptibility to penicillin, will absolutely require MIC determination and TDM in the event of the choice of a glycopeptide and a β-lactam, respectively. In the first situation, to avoid the risk of failure linked to MIC creep, and in the second, to optimize therapeutic choices. In the case of the choice of an antibiotic with better diffusion [81], such as linezolid in cerebral meningeal infections due to coagulase-negative *Staphylococci* [82], we think it is less justified, as the antibiotic concentration obtained at the infection site ensures a priori effectiveness. If the question arises in other clinical situations such as bone and endocardial infections [83], or in microbiological situations for microbial species with multiple resistance mechanisms (natural or acquired), the assay and MIC determination seem to be less justified in the case of pneumonia or primary or secondary bacteremia in a “drainable” source of infection [84].

There are certainly other clinical situations that require TDM, whether in intensive care, where numerous studies have highlighted the risk of therapeutic failure linked to underdosing, or in special populations such as the very obese (risk of underdosing); the elderly with an increased risk of toxicity [85]; and finally patients with cystic fibrosis [86,87] who have treatment difficulties linked to the bacterial species involved (*Pseudomonas aeruginosa*; *Burkholderia cepacia*), to the presence of biofilm, and to an increase in distribution volumes [87]. 

It is important to emphasize that certain complex clinical situations involving changes in renal function and infections with species that are difficult to treat will require plasma assays and MIC measurements. In the case of renal failure in a patient infected by a bacterium with a high MIC, the need to reach the required thresholds may lead to exposure of the patient to increased toxicity [88]. On the other hand, and conversely, in the case of hyper-clearance, an adverse effect may occur with an inability to reach the required thresholds, regardless of the dose administered [62,89]. 

An indication that, in our opinion, will be more and more frequent lies in the use of antibiotics ”concerned by the resistance mechanisms” of the treated species. Over the last ten years, in view of the spread of resistance and to avoid the use of so-called last-resort antibiotics, the possibility of using narrower-spectrum antibiotics based on phenotypic resistance data has become widespread. Until the worldwide spread of ESBL strains, the genotypic character had prevailed. Thus, species producing an enzyme that hydrolyzes β-lactam antibiotics made it impossible to use these β-lactam antibiotics. Concerning ESBL, it was thus unimaginable to use antibiotics that could be hydrolyzed by the enzyme, such as 3GC. Thus, carbapenems, the only antibiotics that cannot be hydrolyzed by ESBL, were the reference antibiotic. More recently, many authors [33,34,90,91,92] have emphasized the possibility of treating ESBL, such as carbapenemase-producing *Enterobacterales,* with a hydrolyzable β-lactam, provided that the concentration at the site of infection meets the PK/PD criteria, i.e., in this particular case, a concentration that is more than 40% of the time in the dosing interval above MIC. It is obvious that therapeutic success as well as the risk of failure in this type of indication depend on the MIC value, as was recently recalled in the MERINO study [92]. 

To conclude, in Table 3 we provide a list of microbiological and clinical situations for which we strongly recommend MIC determination.

## 5. Current Limitations and New Challenges

### 5.1. Limitations Associated with TDM

TDM implies the use of an available, timely, and accurate bioanalytical assay for drug measurements. Many hospital laboratories now routinely measure the most commonly used antibiotics, most often by immunoassay (notably for aminoglycosides and glycopeptides) or by liquid chromatography coupled to ultraviolet or mass-spectrometry detection [93]. However, while immunoanalysis is a common and easy-to-use technique, the use of chromatographic techniques requires expertise and logistics that limit their deployment to a few hospital facilities, which raises the question of the stability of antibiotics during sample transport. Stability studies are available and show that most β-lactams are stable for 6 h at room temperature [94], as well as daptomycin [95], and this period increases to 72 h for fluoroquinolones [96]. After this period, it is recommended to transport the samples at +4 °C within 2 to 3 days [94]. 

Once the plasma concentration is obtained and compared to the MIC, dose adjustments may be necessary. For this there are several strategies, from the most complex to the simplest. In recent years, many precision dosing software programs have been developed to optimize antimicrobial dosing [97]. These programs are based on the use of a population PK model and allow for the identification of patient-specific PK parameters by Bayesian analysis. These programs are generally easy to use, but require two to three samples between two doses to characterize the terminal elimination phase of the antimicrobial +/− its distribution in the patient [98,99]. If a Bayesian model is not available because it does not exist for an antimicrobial or because the patient is too far from the characteristics of the reference population, it is possible to estimate an elimination half-life from two samples in the terminal phase of the curve and to estimate the C_min_, if not available [99]. However, this alternative involves considering a single compartment model with linear elimination and should be used with caution. Finally, in the case of time-dependent antibiotics, a simple trough concentration measurement can identify an exposure < MIC, although this approach cannot be used to calculate patient-specific PK parameters [5]. To date, no study has demonstrated the superiority of the use of Bayesian analyses over much simpler approaches in the field of antibiotic therapy, although knowledge of the patient’s PK parameters may be useful in the case of antimicrobials with non-linear elimination or when the PD objective involves the AUC calculation.

### 5.2. Limitations of MIC Determination 

Determination of the MIC value has a few limitations. First is the time needed to obtain the AST result. The current widely used methods require 2 days and this may extend the duration of inappropriate initial antibiotic therapy and can negatively impact patient outcome, particularly when bacteremia is associated with septic shock [100]. Second, automated AST systems are based on the BMD method and provide results within 6–8 h. However, none of them have the ability to provide accurate MICs [101]. Indeed, panels usually contain only several concentrations of each antimicrobial agent, and the resulting MIC is not always given as an exact value [101]. Recently, Tibbetts et al. reported the first clinical evaluation of the Reveal rapid AST system, tested against randomly selected blood cultures with Gram-negative organisms. The Reveal AST system allows for rapid susceptibility testing of Gram-negative blood cultures, and provides accurate MIC values [102]. Third, reduced antibacterial activity against specific bacterial pathogens at inocula (i.e., elevated MIC or near the breakpoint) above those performed for AST is referred to as the inoculum effect. Cephalosporins and PTZ consistently display observable inoculum effects in vitro, whereas carbapenems are less susceptible to an inoculum effect [103]. The presence of β-lactamase enzymes is the primary mechanism responsible for an inoculum effect, but the observation of an inoculum effect in multiple pathogens lacking β-lactamase enzymes indicates that multiple mechanisms may result in an inoculum effect [103].

## 6. Conclusions

The microbiological and pharmacological tools already available are useful for the treatment optimization and follow-up of serious infections. The implementation of these tests depends on a number of criteria that we tried to list without being exhaustive (Figure 1). We particularly recommend the determination of MIC when organisms have a high level of resistance, when patients present a risk of under-exposure, and in the case of difficult-to-access infections (Table 3). Furthermore, practitioners should not hesitate to use MIC and TDM more widely, without waiting for the appearance of adverse effects or the absence of clinical improvement, especially when the antibiotic chosen is not the best available treatment. In the future, yielding the MIC in less than 24 h and software helping to optimize administration will increase therapeutic adequacy and probably patient survival. Nevertheless, PK-PD data are and will remain tools at the disposal of the clinician, and the patient’s clinical progress is still the most important guide for dose adjustment.

## Figures and Tables

**Table 1:** Microbiological determinants warranting MIC determination according to the EUCAST guidelines [11].

Microbiological Determinants	Bacteria of Concern	Antibiotic of Concern
Agar diffusion method as inappropriate for some antibiotics	Gram-positive bacteria *Staphylococcus* spp. *Enterobacterales*, *P. aeruginosa, A. baumannii*	Daptomycin Dalbavancin Oritavancin Telavancin Vancomycin Teicoplanin Fosfomycin iv Colistin
Absence of detection of the resistance level to β-lactams	*Streptococcus pneumoniae* (reduced susceptibility to penicillin strains) *Haemophilus influenzae* (BLNAR * strains)	β-Lactams
Detection of low-level antibiotic resistance	*Salmonella* sp.	Ciprofloxacin
MIC creep	*Staphylococcus aureus*	Vancomycin
Preserve broad-spectrum antibiotics	*Enterobacterales*	Piperacillin/tazobactam Cephalosporins

* beta-lactamase-negative ampicillin resistance.

**Table 2:** Summary of PK/PD indices associated with efficacy and PK threshold for toxicity for most commonly used antibiotics.

	PK/PD Index	PK/PD Threshold for Efficacy	PK Threshold for Toxicity	
β-Lactams	%*f*T_>MIC_	100% *f*T_>4x MIC_	**Neurotoxicity:** Cefepim: C_min_ > 22 mg/L C_ss_ > 35 mg/L Meropenem: C_min_ > 64 mg/L Piperacillin: C_ss_ > 157 mg/L ^$^ C_ss_ > 360 mg/L **Nephrotoxicity:** Meropenem: C_min_ > 44.5 mg/L Piperacillin: C_min_ > 453 mg/L	[6,44,45,46,47]
Fluoroquinolones	*f*AUC_0–24_/MIC	AUC_0-24_/MIC > 125 C_max_/MIC > 10–12		[48,49]
Aminoglycosides	C_max_/MIC	C_max_/MIC > 8–10	**Oto- and Nephrotoxicity:** Gentamicin, tobramycin: C_min_ > 1 mg/L Amikacin: C_min_ > 5 mg/L	[50,51,52]
Vancomycin	*f*AUC_0–24_/MIC	AUC_0-24_/MIC > 400	**Nephrotoxicity:** C_min_ > 20 mg/L C_ss_ > 25 mg/L	[20,21,53,54]
Linezolid	*f*AUC_0–24_/MIC %*f*T_>MIC_	AUC_0-24_/MIC > 100 85% *f*T > MIC	**Hematotoxicity:** C_min_ > 6 mg/L	[55]
Daptomycin	*f*AUC_0–24_/MIC	AUC_0-24_/MIC > 666	**Myotoxicity:** C_min_ > 24 mg/L	[56]
Colistin	*f*AUC_0–24_/MIC	Unclear	**Nephrotoxicity:** C_min_ > 2.4 mg/L	[57]

C_max_: peak concentration; C_min_: trough concentration; C_ss_: steady-state concentration (when the antibiotic is administered by continuous infusion). ^$^ when used with tazobactam.

**Table 3:** Microbiological and clinical situations for which we strongly recommend MIC determination.

Bacterial resistance	Whenever an alternative to the reference treatment is used	ESBL (e.g., use of tazocilline or cefepime) Carbapenemase (e.g., use of combination of car-bapenems)
Infection site	When antibiotics with limited diffusion are used.	Meningitis (e.g., β-lactams in infection by *S. pneumoniae* or *H. influenzae*)
Patient characteristics	When the population is at risk of under-exposure	Augmented renal clearance (e.g., vancomycin or most β-lactams) Acute care patients
Patient outcome	When the outcome is not favorable	Persistence of bacteremia Unfavorable clinical-laboratory outcome

## Data Availability

Not applicable.
